# Supplementary material for: Adaptive pangenomic remodeling in the Azolla cyanobiont amid a transient microbiome
Source: ISME J. 2025 Jul 29;19(1):wraf154. doi: 10.1093/ismejo/wraf154 (PMC12376041; doi:10.1093/ismejo/wraf154)
Supplement: Supplemental_Methods_Figures_and_Tables_wraf154 [file supplemental_methods_figures_and_tables_wraf154.docx]

Supplemental Information for

**Adaptive pangenomic remodeling in the *Azolla* cyanobiont amid a transient microbiome**

David W. Armitage, Alexandro G. Alonso-Sánchez, Samantha R. Coy, Zhuli Cheng, Arno Hagenbeek, Karla P. López-Martínez, Yong Heng Phua, Alden R. Sears

**SUPPLEMENTAL METHODS**

*Scanning Electron Microscopy*

Ferns were fixed in 2.5% glutaraldehyde for 1 hour at room temperature and then stored in the fridge at 4°C until further processed. The samples were washed thrice with water for 5 minutes each, post-fixed with 2% OsO_4_ then washed again six times in water. They were incubated in 25% and 50% DMSO for 30 minutes each. Samples were split open while submersed in liquid N_2_ with a pre-cooled knife by knocking with a hammer. Split leaves were then transferred to 50% DMSO to warm to room temperature and dehydrated stepwise in ETOH (60%, 70%, 80%, 90%, 100% 3x) for 5 min each step. Samples were then transferred to 50% T-butanol/EtOH for 5 min then to 100% T-butanol. They were frozen at -20°C and freeze-dried in a HITACHI ES-2030 freeze drier, mounted on stubs and sputter coated with gold, and imaged on a Jeol JSM-7900F Scanning Electron Microscope at the OIST Imaging Section.

*Leaf Pocket Enrichment and DNA Extraction*

Once a fern strain had achieved a suitable biomass, we removed approximately 8 grams of plant material from the culture and rinsed it in sterile ultrapure water before removing the roots. Ferns were then agitated for 30s in an Erlenmeyer flask containing 0.2 mL of Triton X-100 in 200 mL of sterile ultrapure water. The contents of this flask were sieved through a flame-sterilized 1 mm steel mesh and then rinsed with sterile ultrapure water for two minutes until all Triton-X had been removed. This material was then divided among the 6 wells of a sterile tissue culture plate containing an enzymatic solution of Cellulase “Ozonuka” R-10 (1 gram), Macerozyme R-10 (0.5 g), Pectinase from *Aspergillus niger* (0.05 g), KH_2_PO_4_ (24 g), Dithiothreitol (0.077 g), and Polyvinylpyrrolidone (0.5 g) dissolved in 50 mL of 0.5M mannitol solution. The plate was placed into a vacuum chamber for tissue infiltration by the enzyme solution for 30-45 minutes at 20 kPa. The solution and plant material were then moved into a 50 mL centrifuge tube and shaken at 32° C at 80 rpm for 18 hours. The resulting macerated *Azolla* material was strained through clean 500 μm Nitex mesh into a sterile glass flask, and material remaining in the mesh was gently rinsed through the mesh with sterile 0.5M mannitol solution. This process flushes the separated leaf pockets into the flask, which, after being allowed to settle, were concentrated by decantation. The leaf pockets were then gently poured into a sterile petri dish and pipetted under a stereo microscope into a single 2 mL microtube containing phosphate buffer solution. This process was repeated for each *Azolla* strain. While our goal was to enrich the proportion of microbial DNA within our samples using the ‘pocket enrichment’ technique described above, numerous host plant cells were also present in our mixture. The cells represented either accompanying hair cells that are the sites of adhesion and nutrient exchange in the *Azolla* leaf pocket, or simply the remaining protoplasts following the enzymatic digest which were pipetted alongside the leaf pockets.

For DNA extraction, leaf pockets collected from each strain were centrifuged and resuspended in 425 μL TE buffer, to which 10 μg/ml RNAse A and 5 mg/mL lysozyme were added. Following a 20 min incubation at 37° C, Proteinase K (100 μg/ml) and 50 μL 10% SDS were added and incubated at 50° C for 2 hours. Next, 250 μL phenol and 250 μL chloroform/isoamyl alcohol (24:1) were added to the tube, mixed gently, and centrifuged at 15000 × g for 2 min. The aqueous supernatant was removed for a second extraction. This step was repeated three times, after which 500 μL chloroform/isoamyl alcohol was added, gently mixed, and centrifuged again for 2 minutes. The aqueous layer was removed and the process was repeated. DNA was then precipitated in cold ethanol and resuspended in 50 μL TE buffer.

*Metagenome processing*

The following table presents software descriptions for all steps of metagenome processing following the JGI metagenome pipeline.

| **Software (version)** | **Purpose** | **Parameters / options** |
| --- | --- | --- |
| RTA 3.4.4 | Illumina base-calling | Default (Illumina) |
| BBDuk  38.79 | Read trimming / decontam | adapter/quality trim; drop reads with ≥4 N, avg Q < 3, length ≤ 51 bp or ≤ 33 %; clip ≥5 G homopolymers |
| BBMap 38.44 | Host & contaminant filter | map to masked refs at 93 % ID; chaff reads removed |
| bbcms 38.44 | Error-correct reads | min k-mer count = 2; high-count fraction = 0.6 |
| metaSPAdes 3.13.0 | Assembly | --meta; kmers 33/55/77/99/127; assembly-only |
| BBMap  38.44 | Read-to-contig mapping | interleaved =true; ambiguous =random; covstats output |
| tRNAscan-SE 2.0.6 | tRNA prediction | modes = bacterial & archaeal; choose better isotype count |
| cmsearch INFERNAL 1.1.3 | rRNA / ncRNA | Rfam 13.0; --cut_tc |
| CRT-CLI 1.2 | CRISPR detection | repeat 20–50 bp; spacer 20–60 bp; window 7 bp; ≥3 repeats |
| Prodigal 2.6.3 | CDS prediction | meta mode; -m (stop at Ns) |
| GeneMarkS-2 1.07 | CDS prediction | --Meta mgm_11.mod; --incomplete_at_gaps 30 |
| LAST lastal 1066 | KO / EC assignment | top-5 hits; ≥2 hits share KO; ≥70 % alignment cover |
| HMMER hmmsearch  3.1b2 | COG, SMART, SUPERFAMILY, CATH, Pfam, TIGRFAM | domE = 0.01 (COG etc.); Pfam --cut_tc; ≥70 % model/protein coverage |
| MetaBAT 2.12.1 | Genome binning | default (coverage + tetranucleotide) |
| CheckM 1.0.12 | Bin QC | default |
| GTDB-tk 0.2.2 | Bin taxonomy | place bins in GTDB reference trees |

**SUPPLEMENTAL FIGURES**

**Fig. S1**. — Schematic overview of the BUSTED-PH approach for phenotype-dependent differential selection. Adapted from <https://github.com/veg/hyphy-analyses/tree/master/BUSTED-PH>. Unrestricted branch-site models estimate different *d*N/*d*S ratios for foreground and background points and can range from 0 to ∞. Constrained models force *d*N/*d*S ≤ 1. These constrained models are then compared to unconstrained models using a likelihood ratio test. Significant test values (*P <* 0.05) support an observed *d*N/*d*S > 1, indicative of positive (diversifying) selection. Nonsignificant values fail to reject the null of *d*N/*d*S ≤ 1, indicative of neutrality or purifying (negative) selection. The final model for differential selection (4) fixes the dN/dS ratio over the entire tree and compares it to model 1, which permits variation in *d*N/*d*S between FG and BG branches. Significant test values support the existence of different dN/dS ratios between focal groups.

**
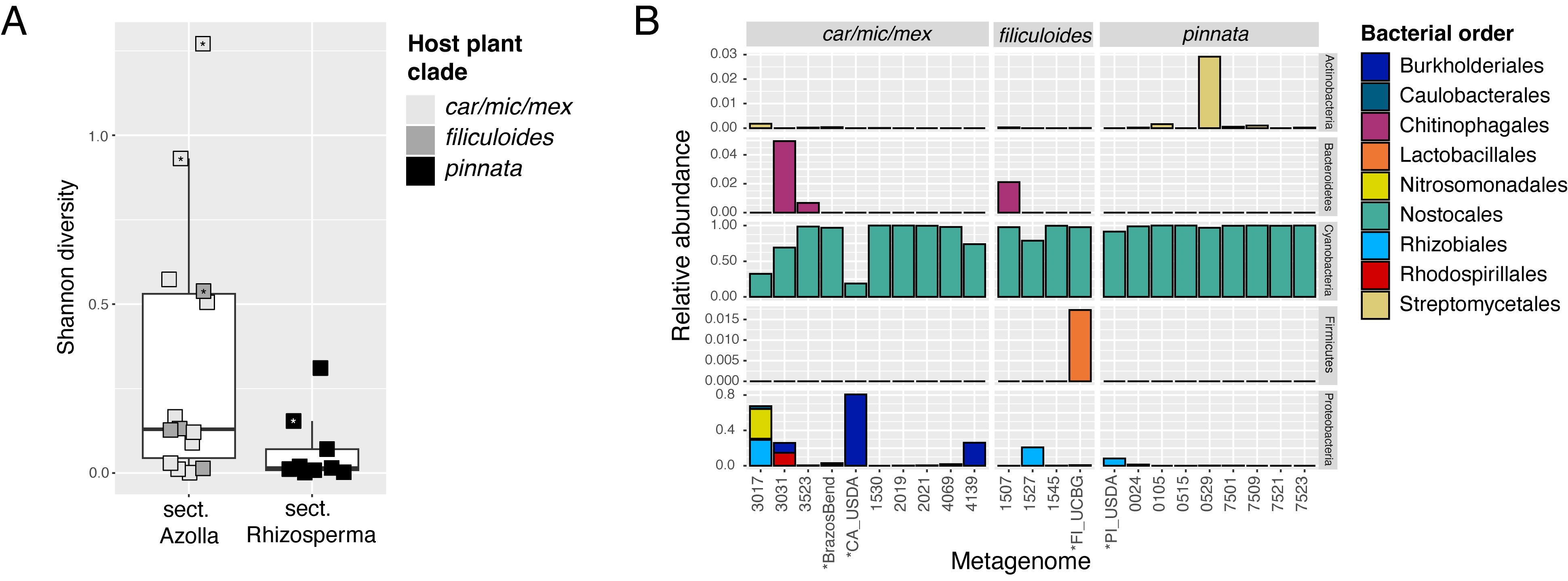
**

**Fig. S2**. — (A) MetaPhlAn-based species diversity estimates for each metagenome. (B) Taxonomic profile of the leaf microbial communities detected in each leaf pocket metagenome. Note the different units for each *y-*axis. Asterisks in points/labels indicate samples collected recently from natural habitats.


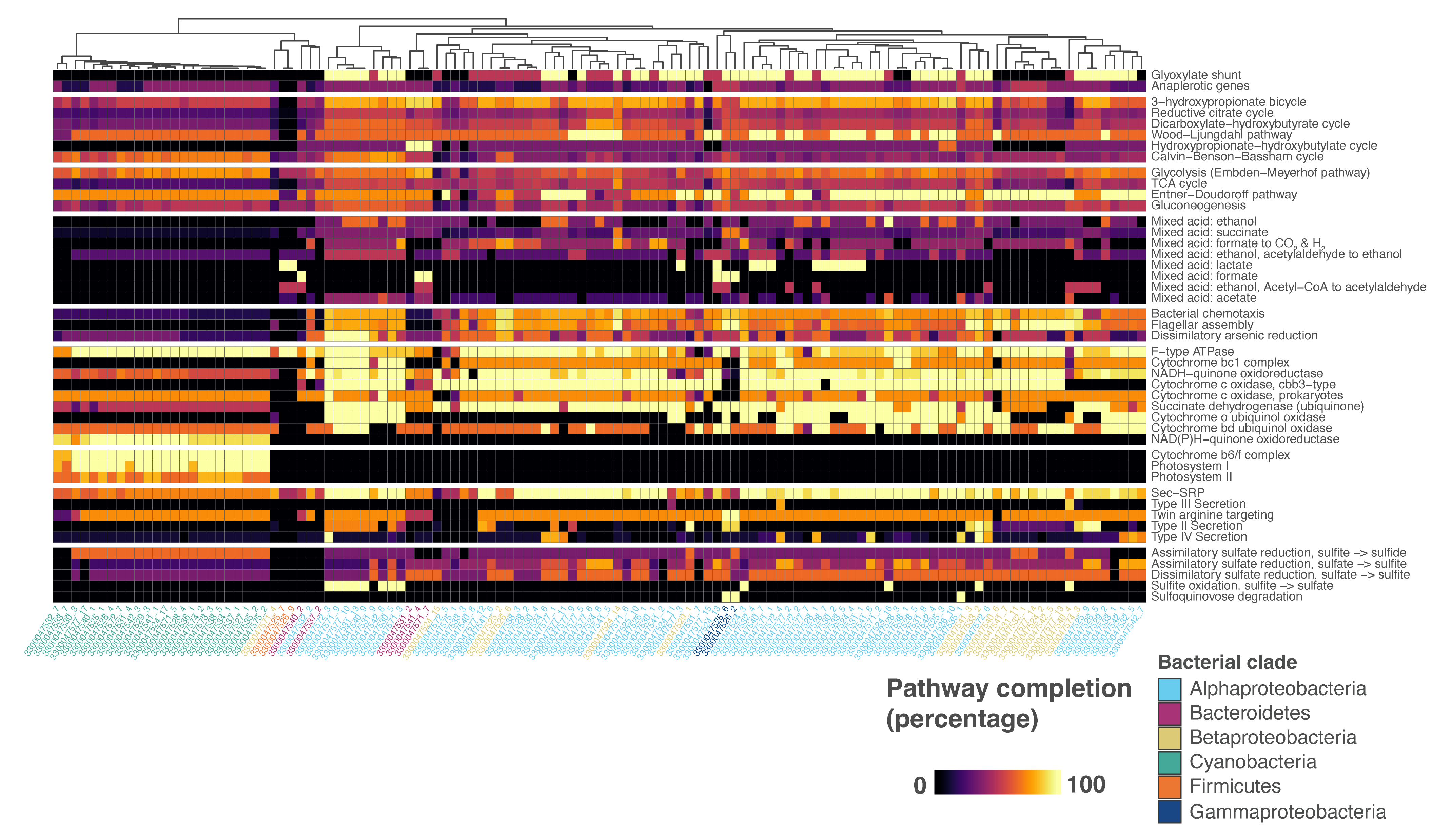


**Fig. S3**. — Heatmap of completeness of key metabolic and physiological pathways (rows) among all MAGs (columns) from the *Azolla* leaf pockets. MAGs are hierarchically clustered by functional pathway similarity following the dendrogram on top. Nitrogen transformation pathways are not pictured as they are already shown in figure 2, but also contribute to the clustering.

**Fig. S4**. — Cophylogeny of *Azolla* plastid marker phylogeny and genomic SNP phylogeny. Bootstrap branch support values greater than 50% are shown.


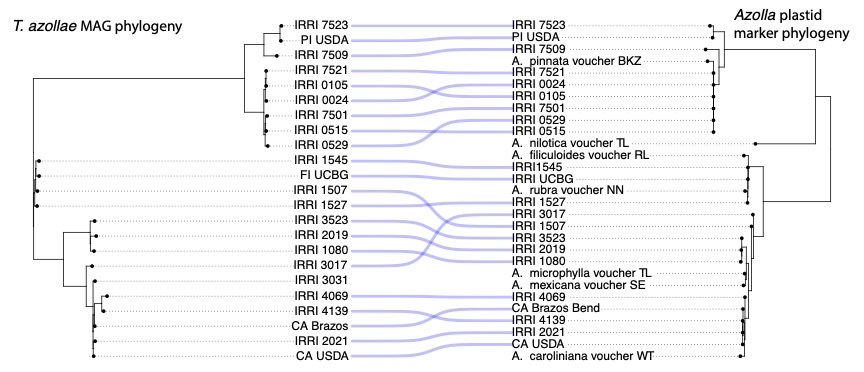


**Fig. S5** — Cophylogeny of *T. azollae* MAG phylogeny and plastid marker phylogeny of the host fern *Azolla*. Voucher specimens are pulled from NCBI Genbank accessions and were not sequenced as part of the present study. Bootstrap support for all *T. azollae* MAG branches is 100%, except for the case of IRRI 7523 and PI USDA, which is 31%.


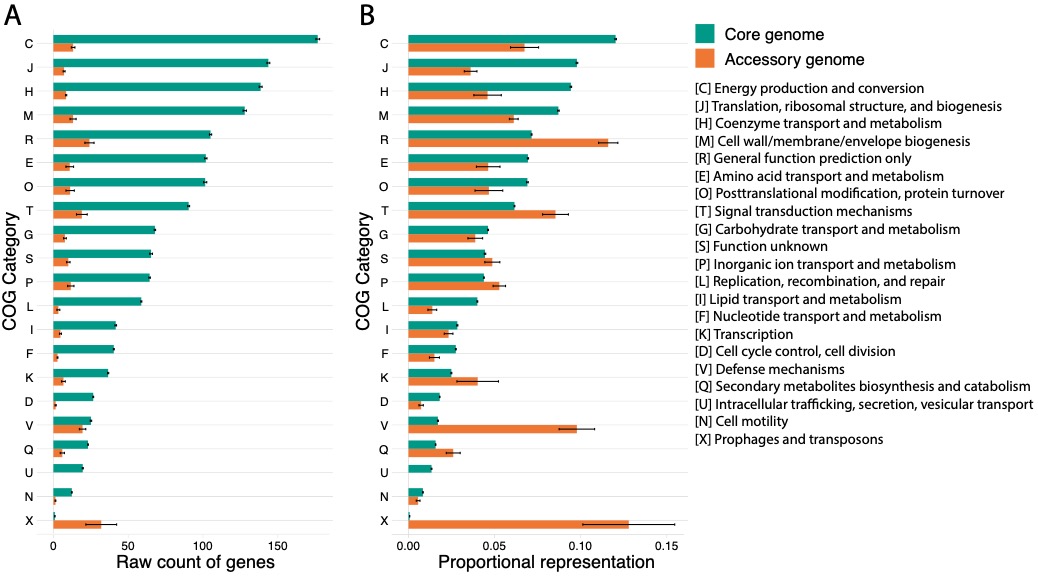


**Fig. S6**. — (A) Raw counts of annotated gene clusters in *T. azollae* sorted into their respective COG categories and location on the *T. azollae* pangenome (see Fig. 3 for core and accessory definitions). (B) Proportional representation of a particular COG category relative to all others within the core or accessory genomes. Here, the accessory genome appears enriched in COG categories T, V, and X.

**Fig. S7**. — Distribution of pseudogene assignment reasons across *T. azollae* MAGs and related reference genomes.
